# Sex, drugs, and early emerging risk: Examining the association between sexual debut and substance use across adolescence

**DOI:** 10.1371/journal.pone.0228432

**Published:** 2020-02-06

**Authors:** D. Angus Clark, M. Brent Donnellan, C. Emily Durbin, Amy K. Nuttall, Brian M. Hicks, Richard W. Robins

**Affiliations:** 1 Department of Psychiatry and Addiction Center, University of Michigan, Ann Arbor, Michigan, United States of America; 2 Department of Psychology, Michigan State University, East Lansing, Michigan, United States of America; 3 Department of Human Development and Family Studies, Michigan State University, East Lansing, Michigan, United States of America; 4 Department of Psychology, University of California, Davis, Davis, California, United States of America; Leibniz Institute for Prevention Research and Epidemiology BIPS, GERMANY

## Abstract

Sexual debut, or first intercourse, predicts problem behaviors such as substance use. This association could reflect a direct effect of debut itself, general developmental trends, or the fact that some youth are more predisposed to a wide array of problem behaviors (e.g., risky sex, substance use). Understanding the association between sexual debut and substance use thus requires methods that can distinguish between these various accounts. In this study the association between sexual debut and substance use was investigated in a longitudinal sample of Mexican-origin youth (N = 674) assessed annually from 5^th^ (M_age_ = 10.86 years, *SD* = 0.51) through 12^th^ grade (M_age_ = 17.69 years, *SD* = 0.48). The longitudinal aspect of the data allowed the direct effect of sexual debut on substance use to be tested while accounting for long-term trends in substance use, and stable individual differences in those trends based on early risk and debut timing. Substance use increased over time, and early risk and debut were consistently associated with more substance use. Sexual debut also modestly predicted an increase in substance use after accounting for these effects, however. Taken together, results provide some evidence consistent with each of the potential explanations for the association between sexual debut and substance use across adolescence.

## Introduction

Sexual debut, or first sexual intercourse, is a normative part of adolescent development [1]. However, sexual debut is also associated with certain problematic outcomes, such as substance use [2–3]. Thus, there is a tension in the literature between recognizing that sexual debut is a normal part of development that should not be pathologized, and understanding why sexual debut is correlated with problem behaviors [3–4]. This issue has important implications—for example, for sex education policies—which increases the need for rigorous evidence about the nature of the association between sexual debut and substance use. Accordingly, we investigated the temporal associations between sexual debut and substance use in a sample of youth followed annually from late childhood until the 12^th^ grade.

### Sexual debut and substance use in adolescence

More than 60% of individuals in the U.S. report their first sexual intercourse between ages 15 and 19 [1, 2, 4, 5, 6]. Similarly, by the end of high school the majority of adolescents (80% - 90%) have used an intoxicating substance, such as alcohol, tobacco, or cannabis [7–8]. In light of the fact that substance use and sexual activity tend to both emerge during adolescence there is ongoing interest in the temporal dynamics between the two behaviors. Substance use and sexual activity are of course complex, distinct adolescent behaviors influenced by a host of personal, familial and sociocultural factors [3, 9], however, there is evidence suggesting that sexual debut itself is associated with subsequent increases in substance use, especially when debut is earlier [10–12].

Broadly speaking there are three primary ways in which sexual debut and substance use could be related over time. First, as noted above, the act of sexual debut could directly predict subsequent increases in substance use [10–12]. For example, once youth become sexually active they may begin to spend more time with other sexually active peers in a social milieu that is more facilitative of substance use [10]. Adolescent sexual activity often does occur in contexts where substances are available [13], and a non-trivial amount of youth report being under the influence of a substance during recent sexual encounters [14].

Second, as youth age they have more opportunities for both sexual activity and substance use, meaning the association between behaviors could largely be an artifact of age-related increases in both behaviors. That is, in adolescence sexual maturity is reached, more time is spent with peers relative to parents, and individuals become increasingly independent (e.g., in later adolescence youth are legally permitted to drive and purchase tobacco products) [15–16]. In the context of these broad developmental shifts increases in both substance use and sexual activity are commonly observed, yet there may not be any more meaningful correspondence between these increases other than the fact they both tend to track a degree of increasing maturity [7, 9, 15].

Finally, the association between sexual debut and substance use could reflect stable individual differences on pre-existing risk factors that contribute to a broad liability for a variety of problem behaviors. Adolescent problem behaviors (e.g., substance use, delinquency, precocious sex) co-occur at high rates, and several theories propose that some youth possess a general tendency toward disinhibition and problem behaviors [17–19]. Thus, the association between sexual debut and substance use, especially earlier in adolescence when sexual activity is rarer, could be due to a non-specific risk for problem behaviors.

Parsing the potential contribution of each possible explanation requires methods that can disaggregate within-person changes from between-person differences. That is, methods that can isolate a direct predictive effect of sexual debut on substance use (i.e., within-person change) by accounting for general age-related trends (i.e., within-person change), and the fact that some youth are more likely to sexually debut and use substances than others (i.e., between-person differences). One such method compares members of twin pairs discordant for sexual debut on substance use to estimate the direct effects of sexual debut while broadly controlling for between-person differences (e.g., shared genetic and environmental risk factors) and age related trends. Findings using this approach suggest that genetic and shared environmental influences largely account for the association between sexual debut and substance use, implying a primarily between-person effect [4, 12, 20, 21, 22].

Despite the strengths of the discordant twin design, they have limitations: they often rely on retrospective reports years after the event, feature only one or two occasions of measurement, are based primarily on European/European-origin samples, and require a sample of many twin pairs meaningfully discordant on the timing of sexual debut. Thus, it is critical to test the generalizability of their conclusions using other methods, particularly longitudinal methods [3, 22, 23, 24]. Longitudinal studies initiated before sexual activity and substance use are relatively normative (e.g., reach 50% prevalence) are particularly useful as it is possible to test the direct effect from sexual debut to substance use while controlling for long-term age-related changes, and long standing between-person differences in those trends [3].

### Present study

The associations between sexual debut and substance use were examined in a sample of Mexican-origin youth assessed annually from 5^th^ through 12^th^ grade. Individual differences in the timing of debut, and childhood variables related to both risky sexual activity and substance use (effortful control, aggressiveness, externalizing problems, peer deviance, and parental monitoring) [3, 4, 25], were also incorporated into the analyses, which allowed the within-person effects of sexual debut to be examined while accounting for between person differences that are related to substance use and adolescent sexual behavior over time. Gender differences were also considered given findings regarding differences that have been observed regarding the timing of sexual debut and rates of substance use [7, 14, 26, 27, 28].

The longitudinal design of this study, paired with the array of risk variables collected at baseline, helps extend and clarify existing research by providing another means of separating out the within- and between-person effects of sexual debut on substance use. Additionally, the focus on Mexican-origin adolescents helps generalize existing findings regarding associations between sexual debut and substance use to a rapidly growing demographic group in the United States [26, 29]. Although trends in sexual activity are largely similar across ethnic groups in the United States [2, 3], Latino youth tend to debut somewhat earlier than European and Asian American youth [30–31]. Latino adolescents may initiate substance use earlier as well [7, 26, 27, 28]. On the other hand, there are distinctive features of Latino culture (e.g., familism) that are associated with reduced risky behavior and that help promote positive youth development [32–33]. Accordingly, it is important to examine whether previously observed ethnic group differences in debut timing, substance use, and cultural traditions also translate into differences in the associations between sexual debut and substance use.

## Method

### Participants and procedures

Participants come from the California Families Project, a longitudinal study of 674 Mexican-origin youth (50.0% girls) and their parents. To recruit participants a simple, unweighted random sample of children was drawn from the rosters of students in the Sacramento and Woodland, CA, school districts. The focal child had to be in the 5^th^ grade, living with their biological mother, and of Mexican origin (i.e., of Mexican ancestry such that either they or previous generations of their family were born in Mexico). Twenty nine percent of focal children were born in Mexico. Participants were interviewed in their homes in Spanish or English, depending on their preference. Parents were not present in the room when their child was interviewed. The first assessment occurred when the youth were in 5^th^ grade (M_age_ = 10.86 years, *SD* = 0.51), and subsequent follow-up assessments were conducted annually until 12^th^ grade (M_age_ = 17.69 years, *SD* = 0.48). Retention rates across waves were high, ranging between 85% and 90% (see Table 1), though due to a data collection programming error substance use was not assessed during the 6^th^ grade assessment for approximately half the sample. All data collection procedures were approved by the University of California, Davis IRB (#217484–22; “Mexican Family Culture and Substance Use Risk and Resilience”); all parents provided informed consent and youths gave their assent.

**Table 1:** Descriptive statistics for substance variables from 5th through 12th grade.

Experimentation	5^th^	6^th^	7^th^	8^th^	9^th^	10^th^	11^th^	12^th^
Full Sample								
M	.06	.11	.30	.70	1.27	1.75	2.35	2.66
SD	.29	.42	.89	1.46	1.95	2.24	2.48	2.56
% No Use	95.60	91.64	83.68	70.68	55.46	45.50	35.33	30.83
N	639	311	576	590	604	589	600	600
Girls								
M	.04	.11	.27	.73	1.31	1.79	2.25	2.56
SD	.21	.44	.81	1.48	1.99	2.29	2.45	2.48
% No Use	97.17	91.45	85.27	69.36	55.74	44.82	36.21	29.84
N	318	152	292	297	305	299	301	305
Boys								
M	.08	.11	.34	.67	1.24	1.70	2.44	2.76
SD	.35	.40	.95	1.44	1.92	2.19	2.50	2.62
% No Use	94.08	91.82	82.04	72.01	55.18	46.21	34.45	31.86
N	321	159	284	293	299	290	299	295
Gender *d*	.14	.00	.08	.04	.04	.04	.08	.08
Frequency of Use	5^th^	6^th^	7^th^	8^th^	9^th^	10^th^	11^th^	12^th^
Full Sample								
M	.04	.07	.15	.40	.66	.94	1.22	1.27
SD	.40	.77	.94	1.50	1.93	2.32	2.95	2.77
% No Use	98.44	97.43	95.30	87.80	81.13	75.00	72.83	68.17
N	639	311	575	590	604	588	600	600
Girls								
M	.03	.13	.13	.46	.70	.82	1.02	.92
SD	.34	1.09	.74	1.61	2.05	2.03	2.73	2.37
% No Use	99.06	96.71	95.21	85.19	81.64	76.51	76.08	73.11
N	318	152	292	297	305	298	301	305
Boys								
M	.06	.02	.18	.33	.62	1.08	1.41	1.62
SD	.45	.14	1.11	1.38	1.80	2.57	3.14	3.09
% No Use	97.82	98.11	95.41	90.44	80.60	73.45	69.57	63.05
N	321	159	283	293	299	290	299	295
Gender *d*	.08	.14	.05	.07	.04	.11	.13	.25
Substance Intentions	5^th^	6^th^	7^th^	8^th^	9^th^	10^th^	11^th^	12^th^
Full Sample								
M	.23	.26	.54	1.25	2.03	2.28	2.69	2.67
SD	1.09	.86	1.74	2.66	3.18	3.43	3.70	3.58
% No Intent	91.09	88.75	83.33	69.49	52.98	49.24	42.00	42.00
N	640	311	576	590	604	589	600	600
Girls								
M	.19	.16	.56	1.49	2.25	2.31	2.57	2.39
SD	1.07	.66	1.62	2.84	3.36	3.46	3.49	3.29
% No Intent	91.54	92.11	82.88	63.97	50.82	49.16	42.19	42.95
N	319	152	292	297	305	299	301	305
Boys								
M	.27	.35	.57	1.00	1.80	2.26	2.81	2.95
SD	1.11	1.01	1.86	2.43	2.96	3.38	3.90	3.83
% No Intent	90.65	85.53	83.80	75.09	55.18	49.31	41.81	41.02
N	321	159	284	293	299	290	299	295
Gender *d*	.07	.22	.01	.19	.14	.01	.06	.16
Accessibility	5^th^	6^th^	7^th^	8^th^	9^th^	10^th^	11^th^	12^th^
Full Sample								
M	1.58	.69	2.12	3.27	6.28	6.55	8.78	8.84
SD	3.16	1.73	3.94	4.90	5.79	6.19	6.03	6.27
% No Access	63.98	78.32	59.54	51.95	28.19	29.54	16.00	17.33
N	633	309	571	589	603	589	600	600
Girls								
M	1.62	.63	2.98	3.87	6.56	6.38	8.66	8.44
SD	3.35	1.73	4.25	5.38	6.13	6.24	6.10	6.52
% No Access	65.51	82.24	60.82	50.84	28.62	34.45	17.61	20.66
N	316	152	291	297	304	299	301	305
Boys								
M	1.54	.74	1.94	2.66	5.98	6.72	8.90	9.25
SD	3.00	1.72	3.58	4.27	5.41	6.11	5.95	5.96
% No Access	62.46	74.52	58.21	53.08	27.76	224.48	14.38	13.90
N	317	157	280	292	299	290	299	295
Gender *d*	.03	.06	.28	.26	.10	.06	.04	.13

M = mean; SD = standard deviation; % No = percentage of youth with scores of 0; N = number of youth reports for a given grade; *d* = Cohen’s *d* for mean difference between girls and boys.

### Measures

#### Sexual debut

An 11-item sexual behavior questionnaire developed for the California Families Project was administered to participants beginning in the 9^th^ grade. For the purposes of this study only a single item was used, “Have you had sexual intercourse during the past 12 months?” Participants responded with either “yes” or “no.” This item was converted into a series of dichotomous “sexual debut” variables by re-coding the initial item such that for a given grade, “yes” responses were only assigned to participants who reported having sexual intercourse during the past 12 months in that grade and no prior grades. This approach resulted in four separate variables that captured five distinct levels of sexual activity initiation: no debut (52.5% of participants), debut reported in 9^th^ grade (11.1%), 10^th^ grade (12.6%), 11^th^ grade (14%), and 12^th^ grade (9.8%). More boys reported debut in 9^th^ grade than girls (40 versus 25), but this gap narrowed over time, with 31 boys and 28 girls reporting debut in 12^th^ grade. In total 153 of 315 boys (49%), and 128 of 316 girls (41%), reported sexual debut at some point during the study.

#### Substance use variables

The psychometric properties of the substance use and early risk questionnaires were evaluated using graded item response models [34]. Although it could be argued that the substance use experimentation and frequency variables (see below) are more consistent with a formative measurement model than the reflective model assumed in item response theory (e.g., lifetime experimentation with substances is a function of its indicators, not vice versa), these models still provide a useful means of holistically quantifying the degree of interrelatedness between items and are thus simply used descriptively to those ends here. Summary statistics from these models are presented in text to provide a concise overview of scale functioning. For ease of interpretation discrimination values are converted to standardized factor loadings, and information values are converted to estimates of reliability [35]. The average reliability estimates presented specifically capture the average information provided by a scale (converted to reliability) between -2 and 2 standard deviations from the mean.

Two substance use variables were measured using the Alcohol, Tobacco, and Other Drug Use scale [36]. *Substance Experimentation* was measured with nine items that assessed whether youth had ever tried a wide range of substances (cannabis, tobacco, hard drugs, and multiple forms of alcohol), and if they ever had experiences becoming intoxicated or getting “high”. Participants responded either “yes” or “no” to each item. Responses were summed to generate a total lifetime experimentation score (possible range between 0 and 9). Items were highly intercorrelated, with the within-wave average factor loadings ranging from λ = .76 to .88 across time. The average reliability between -2 and 2 standard deviations was low in earlier waves, but increased over time, growing from *r*_xx_ = .21 in 5^th^ grade to *r*_xx_ = .62 in 12^th^ grade. The lower reliability values here primarily reflect the fact that 1) in earlier years there was very little substance use (i.e., variability), and 2) the information functions were asymmetric such that reliability was considerably lower below the mean than above. That is, the scale provides more information about youth that use substances than those that use little to no substances. As this is consistent with the aim the scale the asymmetric information functions, and the correspondingly moderate average reliability estimates, are not necessarily problematic here.

*Substance Use Frequency* was measured using nine different items from the Alcohol, Tobacco, and Other Drug Use scale that inquired about the regularity of substance use and intoxication/getting high over the past 3 months. Participants responded a 5 point scale that ranged from 0 (“never”) to 4 (“almost every day”) to each item. Responses across the scale were summed to generate a total substance use frequency score (possible range between 0 and 36). Average within-wave factor loadings ranged from λ = .84 to .95 across time. The average reliability between -2 and 2 standard deviations was low in earlier waves, but increased over time, growing from *r*_xx_ = .05 in 5^th^ grade to *r*_xx_ = .48 in 12^th^ grade. Again, few endorsements in earlier waves and asymmetric information functions contributed to these reliability estimates.

*Substance Use Intentions* were measured using a 9-item scale that assesses willingness to use substances, and plans to use those substances in the next year [37]. Participants responded on either a 3 or 4 point scale that ranged from 0 “*Do not plan to/Definitely will not/Not at all willing”* to either 3 “*Very willing”*, or 4 “*Do plan to/Definitely will*.” Scores for this measure were computed by summing the individual items (possible range between 0 and 33). The average within-wave factor loadings ranged from λ = .85 to .91 across time. The average reliability between -2 and 2 standard deviations was low in earlier waves, but increased over time, growing from *r*_xx_ = .27 in 5^th^ grade to in *r*_xx_ = .68 12^th^ grade. Information functions were again skewed to the left.

Finally, *Access to Substances* was measured using a 7-item scale adapted from the Drug Availability Scale used by the National Household Survey on Drug Abuse [38]. This scale assessed the availability of a range of substances in the youths’ environment. Participants responded on a 4 point scale that ranged from 1 (“extremely difficult/impossible”) to 4 (“extremely easy”). Responses across the scale were summed to generate a total substance use frequency score (possible range between 0 and 21). The average within-wave factor loadings ranged from λ = .84 to .92 across time. The average reliability between -2 and 2 standard deviations was low in earlier waves, but increased over time, growing from *r*_xx_ = .51 in 5^th^ grade to .84 in 12^th^ grade. Information functions were skewed to the left in the earlier grades, but by 9^th^ grade the majority of information was provided with 2 standard deviations from the mean.

#### Early risk

Several measures were used to assess key pre-existing differences between youth on risk factors for problem behaviors [3, 4, 25]. These variables were collected in both 5^th^ and 7^th^ grade, as substance use and sexual activity are rare during this developmental period [3, 7]. *Effortful control* and *trait aggression* were both measured using the Early Adolescent Temperament Questionnaire-Revised [39]. The effortful control scale captures individual differences in activation and inhibitory control (average within-wave factor loadings from λs = .35 to .49 across time and rater; average reliability from *r*_xx_ = .62 to .73 across time and rater), and the aggression scale captures individual differences in hostile actions and hostile reactivity (average within-wave factor loadings from λs = .70 to .74 across time and rater; average reliability from *r*_xx_ = .65 to .84 across time and rater). Mother and self-reports were combined to create composite scale scores. *Externalizing behaviors* were assessed using a standardized psychiatric interview, the Diagnostic Interview Schedule for Children-IV [40]. An externalizing behavior composite was calculated by using the sum of symptoms of conduct disorder and oppositional defiant disorder (symptom counts were correlated at *r* = .39 in 5^th^ grade, and *r* = .21 in 7^th^ grade).

*Peer deviance* was assessed using a 23-item scale adapted from the Delinquent Behavior Scale [41], the Gang Membership Inventory [42], and the Delinquency Scale from the National Youth Survey [43] that measures the degree of deviant behavior, antisocial influence, and gang involvement in the target youth’s peer group (average within-wave factor loadings of λ = .75 and .83; average reliability of *r*_xx_ = .65 and .71). Finally, *parental monitoring* was measured using a 14-item scale that assesses the degree to which parents are aware of their youth’s behavior and various life circumstances [44]. We focused on youth reports of parental monitoring as adolescents’ perception of their parents’ knowledge may be most relevant for their actual behavior [45]. Youth reports of maternal and paternal monitoring efforts were combined into a single parental monitoring variable (average within-wave factor loadings from λ = .67 to .81 across time and parents; average reliability from *r*_xx_ = .83 to .93 across time and parents). Standardized risk composite scores were computed for 5^th^ and 7^th^ grade by calculating the mean of the risk variables following a z-score transformation (effortful control and parental monitoring were reverse scored).

### Data analytic strategy

**Fig 1:**
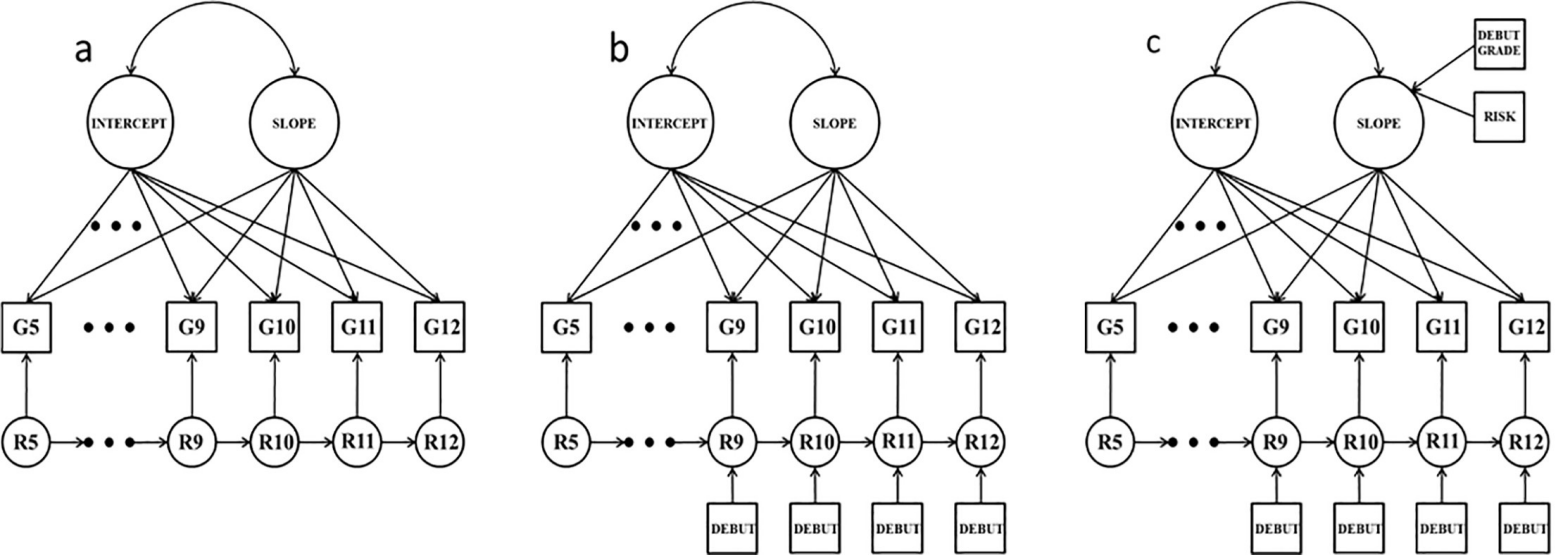
Latent curve models with structured residuals. Panel “a” depicts the unconditional model, panel “b” depicts the time varying covariates model (TVC), and panel “c” depicts the time varying and time invariant covariates model (TVIC). G = substance variable for a grades 5 through 12; R = residual factor. Mean structure and variances/residual variances omitted from figure.

We used latent growth models with structured residuals (LGM-SR; see Fig 1) to disaggregate within- and between-person effects [46–47]. Latent intercept and slope factors were first specified for the observed measurement occasions. The intercept factor captures status at the first time point, and the slope factor captures the rate of change over the course of the study. Each factor also incorporates individual differences across the sample (i.e., intercept and slope variances). A residual structure was then added to the observed variables alongside the latent growth model factors (i.e., each occasion of assessment functioned as an indicator of the growth model and the residual structure). Latent, occasion-specific residual factors were specified that captured deviations between an individual’s observed scores and growth-model implied scores (R5 through R12 in Fig 1). Autoregressive paths linking adjacent latent residual factors were also specified in the residual structure.

For each substance use variable, the optimal unconditional LGM-SR (i.e., a model without any external predictors) was first identified (panel “a” in Fig 1). The slope factor in these models was specified using a latent basis approach in which the first basis coefficient (i.e., slope factor loading) was fixed to 0, while the final basis coefficient was fixed to 1 [48]. The model then freely estimates the intervening basis coefficients, with the mean of the slope factor representing the average amount of change between the first and last observation. Residual factor means were fixed to zero, and residual factor variances were freely estimated. Autoregressive paths also freely varied over time.

**Fig 2:**
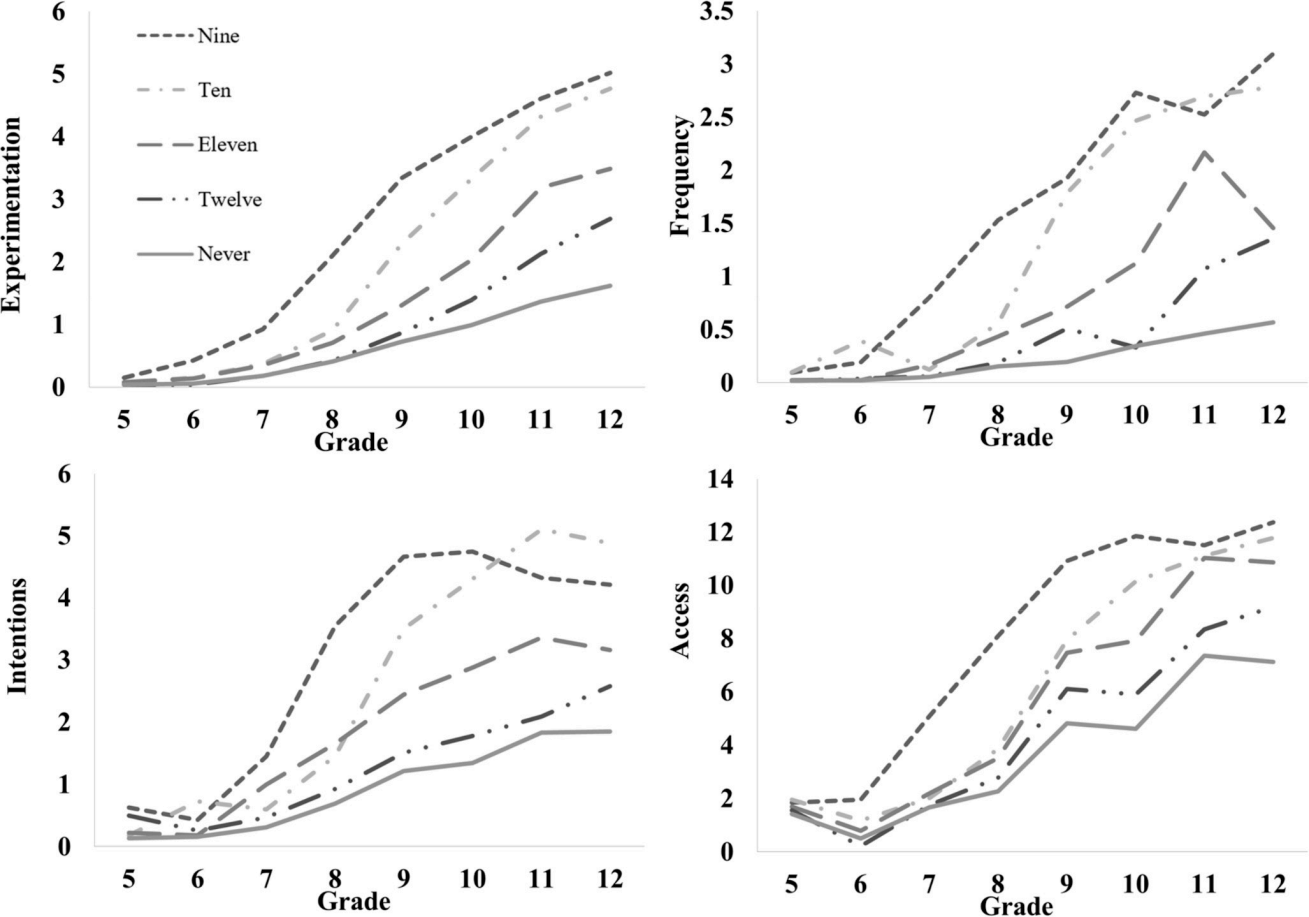
Observed substance trajectories across time as a function of debut timing. Different lines represent trajectories for youth debuting at different grades.

Given that the observed trajectories were markedly non-linear (see Fig 2), the latent basis specification was selected as it provides a parsimonious means of simultaneously capturing general maturational trends as precisely as possible while adjusting for broad individual differences in substance use over time. Notably, the latent basis specification does impose the same shaped growth form on each individual [49]. Spline models have been recommended as an alternative specification to consider because of this, especially when the goal is to compare different growth functions [49]. In the present application however identifying the shape of the growth trajectory per se, and the covariates of that trajectory, was not a major goal. That is, it is well-established that substance use broadly increases from 5^th^ grade through 12^th^ grade—both on average and for most individuals (the trajectories of individuals who consistently abstain would be captured well in both latent basis and spline models)—and the growth model covariates were specifically selected because they are reliable predictors of adolescent substance use in the literature (as is precocious sexual activity). Accordingly, these potentially major limitations of the latent basis specification were somewhat less relevant here.

To identify a more parsimonious model, an iterative series of parameter constraints were tested with the baseline unconditional LGM-SR. More constrained models were compared to their precursors, and differences in fit were examined (change in chi-square, CFI, and RMSEA were all considered). Originally, all autoregressive paths were freely estimated (A1). Next, all autoregressive paths were fixed to equality (A2). If these constraints reduced model fit, then all autoregressive paths in middle school (i.e., grades 5 through 8) and high school (i.e., grades 9 through 12) were separately fixed to equality (A3). If these constraints reduced model fit, all autoregressive paths in middle school were fixed to equality, while autoregressive paths in early (grades 9 and 10) and late (grades 11 and 12) high school were fixed to equality (A4). The A4 specification reflects the fact that late high school is when rates of sexual intercourse and substance use begin to increase more dramatically. If no constraints were supported then all autoregressive paths remained unconstrained in the subsequent models (i.e., A1).

After identifying the optimal unconditional model, sexual debut (i.e., whether youth reported sexual activity for the first time) was added to the residual structure (the time-varying covariates model; TVC) as a predictor of the grades 9 through 12 residual factors (panel “b” in Fig 1). The debut variables serve as quasi time-varying covariates in which participants either never report debut (i.e., “no” at all time points), or are counted as a “yes” at one and only one occasion. Equality in these paths at different grades was tested by constraining paths and comparing fit to the unconstrained model. The four debut variables were then added as predictors of the slope factor (time varying/time invariant covariates model; TVIC), making sexual debut both a time varying, and time invariant, covariate [50]. The early risk variables were also included in the TVIC as predictors of the slope factor (see panel “c” in Fig 1). The time invariant predictors on the slope adjust for stable individual differences in substance use trajectories. This is potentially useful for isolating the direct effect of sexual debut in the residual structure as the sexual debut paths in the TVC could reflect—especially for earlier debuting youth—both time-specific effects, and the fact that youth using more substances across time (who are more likely to debut early) will consistently have more positive residuals given that the unconditional growth model parameters reflect aggregated trends across all youth. Equality constraints were again tested on the paths from debut to the residual factors.

Indeed, the TVIC is particularly informative as it simultaneously captures all three potential accounts for the association between sexual debut and substance use. First, the slope factor mean and variance (SLOPE in Fig 1, panel c) capture the general age-related trends in substance use that track with sexual development in adolescence. Second, the regression paths from debut timing and early risk to the slope factor (DEBUT GRADE and RISK in Fig 1, panel c) capture stable between person differences in substance use, reflecting the extent to which some youth are simply more likely be sexually active and use substances across adolescence. Third, the regression paths from sexual debut to the residual factors (DEBUT in Fig 1, panel c) capture the direct effect of sexual debut, or the extent to which the act of sexual debut in a given grade is associated with increased substance use after conditioning substance use on the general age-related trends in substance use, and stable individual differences in those trends (i.e., the within-person effect of sexual debut after accounting for general maturational trends).

Differences between girls and boys were investigated in the unconditional and conditional models via the comparison of increasingly constrained multigroup models. In these analyses the baseline model was the final model from the full sample analysis (i.e., same pattern of constraints on the residual structure), with estimates allowed to freely estimate across gender. All major analyses were conducted using Mplus version 8.0 [51] with full information maximum likelihood estimation, which provides relatively unbiased parameter estimates in the face of missing data [52]. Confidence intervals were derived via percentile bootstrapping (with 1000 draws), which is particularly effective when estimating confidence intervals with skewed variables such as substance use [53]. If any of the models produced negative variances/residual variances (i.e., “Heywood cases”) these variance estimates were fixed to 0, along with any path emanating from the variable with a negative variance parameter estimate. The data used in the analyses reported here, along with sample syntax files, can be found in the online supplemental material on the Open Science Framework (https://osf.io/95c8w/).

## Results

Descriptive statistics for the substance use variables are reported in Table 1. The average substance use trajectories for youth reporting sexual debut at different times are presented in Fig 2. Earlier debuting youth consistently reported more experimentation, frequency of use, intent to use, and access to substances. Descriptive information for the risk composite variables, based on when youth sexually debuted, is presented in Table 2. Risk scores tended to be higher for youth with earlier sexual debuts.

**Table 2:** Standardized risk in 5th and 7th grade by timing of sexual debut and gender.

	Full Sample			Girls			Boys			
	M	SD	r9	M	SD	r9	M	SD	r9	*d*
5^th^ Grade Risk										
9^th^ Grade Debut	.23	.97	.00	.08	.88	.00	.32	1.03	.00	.25
10^th^ Grade Debut	.12	.74	-.11	.00	.74	-.08	.22	.73	-.10	.30
11^th^ Grade Debut	.10	.64	-.13	.06	.60	-.02	.16	.68	-.16	.16
12^th^ Grade Debut	.06	.64	-.17	.03	.71	-.05	.08	.57	-.24	.08
Never Debuted	-.10	.54	-.33	-.17	.48	-.25	-.02	.59	-.34	.28
7^th^ Grade Risk										
9^th^ Grade Debut	.43	.94	.00	.27	.74	.00	.52	1.03	.00	.28
10^th^ Grade Debut	.19	.66	-.24	-.11	.58	-.38	.41	.63	-.11	.86
11^th^ Grade Debut	.07	.66	-.36	.19	.76	-.08	-.08	.49	-.60	.42
12^th^ Grade Debut	.00	.60	-.43	.09	.56	-.18	-.11	.63	-.63	.36
Never Debuted	-.14	.56	-.57	-.20	.51	-.47	-.07	.60	-.59	.23

M = Mean; SD = Standard Deviation; r9 = risk scores in reference to youth debuting in 9^th^ grade; *d* = Cohen’s *d* for mean difference between girls and boys.

### Substance experimentation

The unconditional model (A3; see supplemental material for full fit information across models: https://osf.io/95c8w/) fit the data at: *χ*^*2*^ = 17.88, *df* = 24, *p* = .81; RMSEA = .000; SRMR = .021; CFI = 1.00; TLI = 1.00. Parameter estimates are reported in Table 3 (see supplemental materials for unconditional multi-group model results for girls and boys: https://osf.io/95c8w/). In this and subsequent substance experimentation models the intercept factor variance was fixed to zero as it was trivial in magnitude and led to non-positive definite solutions when freely estimated. Results from the TVC and TVIC models can be found in Table 4. In both models it appeared that the sexual debut paths in the residual structure could be fixed to equality across time without a notable decline in model fit (Δ*χ*^*2*^ of 10.33 and 3.89; Δdf = 3; ΔRMSEA of -.001 and -.001; ΔCFIs of -.003 and -.001). Sexual debut was associated with a small but statistically significant positive effect in the TVC (b = .51; 95% CI: .31, .71), though the chi square difference test did suggest that the effect of 12^th^ grade debut may be smaller than the effect of debut in earlier grades. The effect of sexual debut on the residual structure remained statistically significant in the TVIC—that is, after adjusting the growth part of the model for stable individual differences in substance use trajectories based on early risk and debut timing—though the unstandardized path estimate was 37% smaller (b = .33; 95% CI: .12, .51). Sexual debut in 9^th^, 10^th^, and 11^th^ grade, and 7^th^ grade risk, were all significantly associated with more substance experimentation over time in the TVIC (Table 4). Neither the time varying (Δ*χ*^*2*^ = .14; Δdf = 1; ΔRMSEA = .000; ΔCFI = .000) nor time invariant (Δ*χ*^*2*^ = 11.33; Δdf = 5; ΔRMSEA = .000; ΔCFI = -.001) effects differed across boys and girls.

**Table 3:** Unstandardized parameter estimates from unconditional models.

Parameter	Experimentation	Frequency of Use	Substance Intentions	Accessibility
Intercept Mean	.06*	.03*	.20*	1.41*
Intercept Variance	.00	.01	.52	4.59*
Slope Mean	2.66*	1.22*	2.46*	7.42*
Slope Variance	1.89	3.19*	7.53*	29.50*
Intercept-Slope Covariance	.00	.13*	-.44	-.17
Basis Coefficients				
5^th^ Grade	.00	.00	.00	.00
6^th^ Grade	.03*	.05*	.04	-.08*
7^th^ Grade	.09*	.10*	.16*	.12*
8^th^ Grade	.24*	.33*	.45*	.26*
9^th^ Grade	.46*	.49*	.73*	.63*
10^th^ Grade	.64*	.83*	.88*	.71*
11^th^ Grade	.86*	.92*	1.02*	.99*
12^th^ Grade	1.00	1.00	1.00	1.00
Autoregressive Coefficients				
5^th^→6^th^ Grade	1.17*	2.11*	-.38	-.28
6^th^→7^th^ Grade	1.17*	.27*	-.12	.97
7^th^→8^th^ Grade	1.17*	.79*	.72*	.55*
8^th^→9^th^ Grade	.97*	.19*	.33*	.42*
9^th^→10^th^ Grade	.97*	.36*	.38*	.41*
10^th^→11^th^ Grade	.90*	.46*	.19	-.02
11^th^→12^th^ Grade	.90*	.36*	.28*	-1.26

* = *p* < .05. Unstandardized parameter estimates presented.

**Table 4:** Effects of sexual debut on the residual structure and slope factor.

	Experimentation			Frequency of Use			Substance Intentions			Accessibility		
	b	95% CI	β	b	95% CI	β	b	95% CI	β	b	95% CI	β
TVC												
9^th^ Grade Debut	.51	[.31, .71]*	.09	.69	[.28, 1.17]*	.13	.59	[.15, 1.08]*	.08	.88	[.36, 1.49]*	.07
10^th^ Grade Debut	.51	[.31, .71]*	.08	.69	[.28, 1.17]*	.13	.59	[.15, 1.08]*	.08	.88	[.36, 1.49]*	.08
11^th^ Grade Debut	.51	[.31, .71]*	.07	.69	[.28, 1.17]*	.09	.59	[.15, 1.08]*	.08	.88	[.36, 1.49]*	.13
12^th^ Grade Debut	.51	[.31, .71]*	.07	.69	[.28, 1.17]*	.10	.59	[.15, 1.08]*	.08	.88	[.36, 1.49]*	.11
TVIC												
*Residual Paths*												
9^th^ Grade Debut	.33	[.12, .51]*	.06	.37	[.05, .76]*	.08	.41	[.02, .83]*	.08	.77	[.32, 1.23]*	.06
10^th^ Grade Debut	.33	[.12, .51]*	.06	.37	[.05, .76]*	.07	.41	[.02, .83]*	.08	.77	[.32, 1.23]*	.07
11^th^ Grade Debut	.33	[.12, .51]*	.05	.37	[.05, .76]*	.05	.41	[.02, .83]*	.08	.77	[.32, 1.23]*	.10
12^th^ Grade Debut	.33	[.12, .51]*	.05	.37	[.05, .76]*	.06	.41	[.02, .83]*	.08	.77	[.32, 1.23]*	.09
*Slope Factor Paths*												
9^th^ Grade Debut	2.55	[1.83, 3.27]*	.58	2.19	[1.00, 3.16]*	.42	2.37	[1.40, 3.40]*	.28	3.34	[1.75, 5.17]*	.21
10^th^ Grade Debut	2.56	[1.87, 3.28]*	.61	1.56	[.57, 2.57]*	.31	2.08	[1.19, 3.08]*	.26	3.41	[1.90, 4.88]*	.22
11^th^ Grade Debut	1.34	[.69, 1.99]*	.33	.63	[.12, 1.14]*	.13	1.02	[.38, 1.73]*	.13	2.61	[1.08, 4.06]*	.17
12^th^ Grade Debut	.34	[-.29, 1.01]	.08	.09	[-.28, .43]	.18	-.03	[-.61, .45]	.00	.00	[-1.74, 1.73]	.00
5^th^ Grade Risk	.02	[-.34, .36]	.01	-.18	[-.48, .25]	-.07	.06	[-.41, .56]	.02	-.38	[-1.19, .39]	-.05
7^th^ Grade Risk	1.03	[61, 1.46]*	.49	.87	[.34, 1.30]*	.35	1.61	[1.03, 2.10]*	.40	1.56	[.69, 2.44]*	.20

TVC = time varying covariates model; TVIC time varying and time invariant covariates model; Residual Paths = paths from sexual debut to residual structure in TVIC; Slope Factor Paths = paths from sexual debut and early risk to slope factor in TVIC; b = unstandardized regression coefficients; 95% CI = 95% confidence intervals based on percentile bootstrap procedure (1000 draws); β = standardized regression coefficients.

* = 95% confidence interval does not include .00.

### Substance use frequency

The unconditional model (A1; see S1) fit the data at: *χ*^*2*^ = 101.04, *df* = 18, *p* < .001; RMSEA = .083; SRMR = .064; CFI = .954; TLI = .928. Parameter estimates are reported in Table 3. Results for the TVC and TVIC are reported in Table 4. In both models it appeared that the sexual debut paths in the residual structure could be fixed to equality across time without a notable decline in model fit (Δ*χ*^*2*^ of 6.77 and 11.45; Δdf = 3; ΔRMSEA of -.003 and -.002; ΔCFI of -.003 to -.004). Sexual debut was a modest but statistically significant predictor of the residual factors in the TVC (b = .69; 95% CI: .18, 1.06). This effect remained statistically significant in the TVIC, however, the unstandardized path estimate was reduced by 46% (b = .37; 95% CI: .05, .76), and the chi square difference test suggested that effects may be stronger for youth debuting in 11^th^ and 12^th^ grade compared to those that debuted earlier. Sexual debut in 9^th^ and 10^th^ grade, and 7^th^ grade risk, were all significantly associated with more frequent substance use over time in the TVIC. The multi-group TVIC for examining gender differences encountered convergence problems, so models were estimated separately for girls and boys. The effect of debut in the residual structure was statistically significant for both sexes and in the same direction, though the path estimate did appear somewhat larger for boys than girls (b of .72 versus .39).

### Substance use intentions

The unconditional model (A1; see S1) fit the data at: *χ*^*2*^ = 32.01, *df* = 18, *p* = .02; RMSEA = .034; SRMR = .030; CFI = .990; TLI = .985. Parameter estimates are reported in Table 3. Results for the TVC and TVIC are reported in Table 4. In both models the sexual debut paths in the residual structure could be fixed to equality across time without a notable decline in model fit (Δ*χ*^*2*^ of 6.88 and .89; Δdf = 3; ΔRMSEA of -.002 and -.003; ΔCFI from -.002 to +.002). Sexual debut was a small but statistically significant predictor of the residual structure in the TVC (b = .59; 95% CI: .15, 1.08). The effect of sexual debut in the residual structure remained statistically significant in the TVIC, with the unstandardized path reduced by 30% (b = .41; 95% CI: .02, .83). Sexual debut in 9^th^ and 10^th^ grade, and 7^th^ grade risk, were all significantly associated with greater substance use intentions over time in the TVIC. Neither time varying (Δ*χ*^*2*^ = .03; Δdf = 1; ΔRMSEA = -.001; ΔCFI = +.001) nor time invariant (Δ*χ*^*2*^ = 13.06; Δdf = 6; ΔRMSEA = -.001; ΔCFI = -.003) effects appeared to differ substantially across boys and girls, though the chi square difference test did suggest that the effect of 11^th^ grade debut on the slope factor may be larger for girls than boys (b of 1.20 versus .45).

### Access to substances

The unconditional model (A1; S1) fit the data at: *χ*^*2*^ = 58.68, *df* = 18, *p* < .001; RMSEA = .058; SRMR = .048; CFI = .981; TLI = .971. Parameter estimates are reported in Table 3. Results for the TVC and TVIC are reported in Table 4. In both models it appeared that the sexual debut paths in the residual structure could be fixed to equality across time without a notable decline in model fit (Δ*χ*^*2*^ of 14.01 and 5.57; Δdf of 3 and 5; ΔRMSEA of .00 and -.003; ΔCFI of -.004 and +.010; in the constrained TVIC the residual variance for the 6^th^ grade residual factor was negative, and so it and its autoregressive path to the 7^th^ grade residual factor were fixed to 0). There was a small but significant effect of sexual debut on the residual factors in the TVC (b = .88; 95% CI: .36, 1.49), though the chi square difference test did suggest that the effects of 11^th^ and 12^th^ grade debut may be smaller than the effects of debut in earlier grades. The effect of debut in the residual structure remained significant in the TVIC, with the unstandardized path reduced by 12% (b = .77; 95% CI: .32, 1.23). Sexual debut in 9^th^ and 10^th^ grade, and 7^th^ grade risk, were all significantly associated with greater substance use intentions over time in the TVIC. Neither time varying (Δ*χ*^*2*^ = .73; Δdf = 1; ΔRMSEA = -.001; ΔCFI = .000) nor time invariant (Δ*χ*^*2*^ = 4.50; Δdf = 6; ΔRMSEA = -.002; ΔCFI = +.001) effects differed across boys and girls.

## Discussion

The association between sexual debut and substance use was examined at different levels of analysis using data from a sample of youth followed annually from the 5^th^ through the 12^th^ grade. Consistent with previous findings, youth with earlier sexual debuts reported more substance use across adolescence than youth who debuted later or not at all during the course of the study. Youth scoring higher on early risk factors also demonstrated higher levels of substance use across time, and were more likely to sexually debut earlier. After accounting for these risk factors for increased substance use (and problem behaviors broadly) across time, and general age-related increases in substance use, sexual debut was a modest within-person predictor of greater substance experimentation, frequency of use, intent to use, and access to substances. Effect sizes were small by conventional standards, but the size and direction of effects were consistent across the substance variables. Consistent gender differences were not detected in these effects.

Thus, there was evidence for both between- and within-person associations between sexual debut and substance use. Results reinforce the importance of a general risk for adolescent problem behavior while tentatively suggesting that the act of sexual debut itself may entail some additional risk. The amount of variance in substance use that can be isolated and attributed to debut itself though is likely modest, especially relative to the between-person effects regarding prior risk factors. Early debuting youth consistently reported more use across time than later or never debuting youth, and many of these trends were evident even before sexual intercourse is more typical (i.e., earlier than 8^th^ grade). Higher scores on late childhood predictors of substance use and risky sexual activity were similarly related to more substance use over time, especially when measured in 7^th^ grade (as compared to 5^th^ grade). Results therefore support twin studies suggesting that associations between sexual debut and delinquent behaviors are largely between-person. It is notable though that some within-person debut effects emerged after controlling for general age-related trends, and the large between person longitudinal trends. Future research can attempt to more precisely highlight the factors accounting for this, albeit modest, predictive trend.

### Limitations

The study had several limitations. The sexual behaviors questionnaire was only included in the study protocol beginning in the 9^th^ grade because of concerns about the sensitivity of such questions at earlier ages. Consequently, there was low sensitivity to detect youth who initiated before the 8^th^ or 9^th^ grade. This was likely a small group, however, as sexual debut before high school is uncommon in the general population [14, 31]. Further, in this sample only 11% (*n* = 65) of youth reported sexual activity in the 9^th^ grade, suggesting less than 11% of the sample initiated sexual activity before high school. The 12-month reporting period of the questionnaire also means that some reports of sexual debut in 9^th^ grade could have occurred the previous year (i.e., 8^th^ grade). Relatedly, no item included in the sexual behaviors questionnaire directly asked about sexual debut. Instead, the item used simply inquired about sexual activity in the past year so that sexual debut could only be considered on an annual time scale. Although this broad time scale precludes the examination of more precise temporal trends, it does allow for the investigation of general patterns over time. Moreover, although this issue may be especially problematic for the lifetime experimentation variable, the other substance variables center on more immediate or prospective behaviors, meaning they are more likely to capture post-debut behaviors and sentiments.

Further, although the sexual debut item used specifically inquired about sexual intercourse, the term “sexual intercourse” may be somewhat ambiguous to youth [2]. However, the interview formant may have allowed for clarification of any ambiguities. A potential drawback of an interview approach is that youth may have been less willing to disclose about sensitive topics relative to a more anonymous collection format. However, youth were allowed to enter their own responses to these questions anonymously on a laptop if they wished.

There were also some limitations in the analyses. Substance use variables were sometimes skewed, especially in earlier grades, and may have been better modeled using zero inflated count models [54]. Unfortunately, serious convergence issues were encountered when estimating such models. Although less than ideal, one of the primary issues with skewed data is that standard errors may be biased, not the parameter estimates per se. The consistency of results across multiple variables and analyses helps protect against concerns of capitalizing on chance [55], especially given that some variables (e.g., substance use intentions) were less skewed than others (e.g., substance use frequency). Further, percentile bootstrapped confidence intervals tend to perform well in the presence of non-normality [53, 56].

Finally, the focus here was primarily on the conceptual path running from sexual debut to substance use. We emphasized this path because much of the interest in the relevant literature is specifically about sexual debut itself as a particularly noteworthy developmental milestone that may promote various risky behaviors. However, sexual activity is often reported as occurring in situations with concurrent substance use (e.g., parties) and many youth report being under the influence of some substance during their most recent sexual encounter [13–14]. That is, substance use and sexual activity are tightly linked in many adolescent contexts, and so it is likely that associations between sexual activity and substance are bi-directional such that substance use may increase the likelihood of sexual activity, which in turns promotes an increase in substance use, etc. Future work should build on the present and related findings by exploring the bi-directional dynamics between substance use and sexual activity (especially sexual behaviors beyond simple debut) across adolescence.

## Conclusion

Sexual debut was modestly but consistently associated with greater substance use, even after accounting for normative age-related increases in substance use during adolescence, and the fact that earlier debuting youth consistently reported more substance use. Results imply associations between sexual debut and substance use at both the within- and between-person level. Some youth consistently use more substances and are more sexually active than their peers (between-person effects), but substance use and sexual activity also become more widespread across adolescence in general (within-person effect), and sexual debut predicts a slight increase in substance use over and above these other two trends (within-person effect). Understanding the association between sexual debut and substance use is helpful to identify at-risk youth and problematic behaviors for intervention without broadly stigmatizing adolescent sexual activity [4].

## References

[1] Halpern C. T., & Haydon A. A. (2012). Sexual timetables for oral-genital, vaginal, and anal intercourse: Sociodemographic comparisons in a nationally representative sample of adolescents. Research and Practice, 102(6), 1221–1228. 10.2105/AJPH.2011.300394 PMC3394539 22571710

[2] Savin-Williams R. C., & Diamond L. M. (2004). Sex In Lerner R. M. & Steinberg L. (Eds.), Handbook of Adolescent Psychology (pp. 139–282). Hoboken, NJ: John Wiley & Sons, Inc.

[3] Zimmer-Gembeck M. J., & Helfand M. (2008). Ten years of longitudinal research on U.S. adolescent sexual behavior: Developmental correlates of sexual intercourse, and the importance of age, gender and ethnic background. Developmental Review, 28, 153–224.

[4] Harden K. P. (2014). Genetic influences on adolescent sexual behavior: Why genes matter for environmentally oriented researchers. Psychological Bulletin, 140(2), 434–465. 10.1037/a0033564 23855958 PMC3893311

[5] Bozon M. (2003). At what age do women and men have their first sexual intercourse? World comparisons and recent trends. Population and Societies, 391, 1–4.

[6] Haydon A. A., Herring A. H., Prinstein M. J., & Halpern C. T. (2012). Beyond age at first sex: Patterns of emerging sexual behavior in adolescence and young adulthood. Journal of Adolescent Health, 50, 456–463. 10.1016/j.jadohealth.2011.09.006 22525108 PMC3336094

[7] Chassin L., Hussong A., Barrera M., Molina B. S. G., Trim R., & Ritter J. (2004), Adolescent substance use In Lerner R. M. & Steinberg L. (Eds.), Handbook of Adolescent Psychology (pp. 139–282). Hoboken, NJ: John Wiley & Sons, Inc.

[8] Young S. E., Corley R. P., Stallings M. C., Rhee S. H., Crowley T. J., & Hewitt J. K. (2002). Substance use, abuse and dependence in adolescence: Prevalence, symptom profiles and correlations. Drug and Alcohol Dependence, 68, 309–322. 10.1016/s0376-8716(02)00225-9 12393225

[9] Windle M., & Windle R. C. (2003). Alcohol and other substance use and abuse In Adams G. R. & Berzonsky M. D. (Eds.), Blackwell Handbooks of Developmental Psychology. Blackwell Handbook of Adolescence (pp. 450–469). Malden, Blackwell Publishing.

[10] Armor T., & Haynie D. L. (2007). Adolescent sexual debut and later delinquency. Journal of Youth and Adolescence, 36, 141–152.

[11] Cornelius J. R., Clark D. B., Reynolds M., Kirisci L., & Tarter R. (2007). Early age of first sexual intercourse and affiliation with deviant peers predict development of SUD: A prospective longitudinal study. Addictive Behaviors, 32, 850–854. 10.1016/j.addbeh.2006.06.027 16839696

[12] Donahue K. L., Lichtenstein P., Langstrom N., & D’Onofrio B. M. (2013). Why does early sexual intercourse predict subsequent maladjustment? Exploring potential familial confounds. Health Psychology, 32(2), 180–189. 10.1037/a0028922 22708520 PMC3664184

[13] O’Sullivan L. F., & Thompson A. E. (2014). Sexuality in adolescence. APA Handbook of Sexuality and Psychology, 1, 433–486.

[14] Centers for Disease Control and Prevention. (2017). Youth Risk Behavior Survey Data. Available at: www.cdc.gov/yrbs. Accessed on 11/05/2018.

[15] Hensel D. J., & Fortenberry J. D. (2014). Life-span sexuality through a sexual health perspective. APA Handbook of Sexuality and Psychology, 1, 385–413.

[16] Zimmer-Gembeck M. J., & Collins W. A. (2003). Autonomy development during adolescence In Adams G. R. & Berzonsky M. D. (Eds.), Blackwell Handbooks of Developmental Psychology. Blackwell Handbook of Adolescence (pp. 175–204). Malden, Blackwell Publishing.

[17] Jessor R. (2014). Problem Behavior Theory: A half century of research on adolescent behavior and development In Lerner R. M., Petersen A. C., Silbereisen R.K., & Brooks-Gunn J.(Eds.)."The developmental science of adolescence: History through autobiography." New York: Psychology Press, 239–256.

[18] Krueger R. F. (2002). Personality from a realist’s perspective: Personality traits, criminal behaviors, and the externalizing spectrum. Journal of Research in Personality, 36(6), 564–572.

[19] Moffitt T. E. (1993). Adolescence-limited and life-course-persistent antisocial behavior: A developmental taxonomy. Psychological Review, 100(4), 674–701. 8255953

[20] Deutsch A. R., Slutske W. S., Heath A. C., Madden P. A. F., & Martin N. G. (2014). Substance use and sexual intercourse onsets in adolescence: A genetically informative discordant twin design. Journal of Adolescent Health, 54, 114–116. 10.1016/j.jadohealth.2013.07.013 23992762 PMC3872214

[21] Harden K. P., Mendle J., Hill J. E., Turkheimer E., & Emery R. E. (2008). Rethinking timing of first sex and delinquency. Journal of Youth and Adolescence, 37, 373–385. 10.1007/s10964-007-9228-9 21479148 PMC3071511

[22] Johnson W., Turkeimer E., Gottesman I. I., & Bouchard T. J. (2009). Beyond heritability: Twin studies in behavioral research. Current Directions in Psychological Science, 18 (4), 217–220. 10.1111/j.1467-8721.2009.01639.x PMC2899491 20625474

[23] Barnes J. C., & Boutwell B. B. (2013). A demonstration of the generalizability of twin-based research on antisocial behavior. Behavioral Genetics, 43(2), 120–131. 10.1007/s10519-012-9580-8 PMC3683969 23274656

[24] D’Onofrio B. M., Lahey B. B., Turkheimer E., & Lichtenstein P. (2013). Critical need for family-based, quasi-experimental designs in integrating genetic and social science research. American Journal of Public Health, 103(1), 46–55. 10.2105/AJPH.2013.301252 PMC3778076 23927516

[25] Conduct Problems Prevention Research Group. (2014). Trajectories of risk for early sexual activity and early substance use in the fast track prevention program. Prevention Science, 15(1), 33–46. 10.1007/s11121-012-0328-8 PMC3883936 23417666

[26] Chen, P., & Jacobson, K. C. (2012). Developmental trajectories of substance use from early adolescence to young adulthood: Gender and racial/ethnic differences. 10.1016/j.jadohealth.2011.05.013 PMC3264901 22265111

[27] Simoes C., Matos M. G., Batista-Foguet J. M., & Simons-Morton B. (2014). Substance use across adolescence: Do gender and age matter? Psicologia: Reflexao e Critica, 27(1),179–188.

[28] Young S. E., Corley R. P., Stallings M. C., Rhee S. H., Crowley T. J., & Hewitt J. K. (2002). Substance use, abuse and dependence in adolescence: Prevalence, symptom profiles and correlations. Drug and Alcohol Dependence, 68, 309–322. 10.1016/s0376-8716(02)00225-9 12393225

[29] U.S. Census. (2011). Overview of race and Hispanic origin: 2010.

[30] Biello K. B., Ickovics J., Niccolai L., Lin H., & Kershaw T. (2013). Racial differences in age at first sexual intercourse: Residential racial segregation and the black-white disparity among U.S. adolescents. Public Health Reports, 128(1), 23–32. 23450882 10.1177/00333549131282S103 PMC3562743

[31] Cavazos-Rehg P. A., Krauss M. J., Spitznagel E. L., Schootman M., Bucholz K. K., … & Bierut L. J. (2009). Age of sexual debut among US adolescents. Contraception, 80(2), 158–162. 10.1016/j.contraception.2009.02.014 19631791 PMC3064497

[32] Dumka L. E., Gonzales N. A., Bonds D. D., & Millsap R. E. (2009). Academic success of Mexican origin adolescent boys and girls: The role of mothers’ and fathers’ parenting and cultural orientation. Sex roles, 60(7–8), 588 10.1007/s11199-008-9518-z 21731172 PMC3128498

[33] Wheeler L. A., Zeiders K. H., Updegraff K. A., Umaña-Taylor A. J., de Jésus R., Sue A., et al (2017). Mexican-origin youth’s risk behavior from adolescence to young adulthood: The role of familism values. Developmental psychology, 53(1), 126 10.1037/dev0000251 28026193 PMC5198906

[34] Samejima F. (1969). Estimation of latent ability using a response pattern of graded scores. Psychometrika monograph supplement.

[35] Thissen D., & Orlando M. (2001). Item response theory for items scored in two categories In Test scoring (pp. 85–152). Routledge.

[36] Elliott D.S., Huizinga D., & Ageton S.S. (1985). Explaining delinquency and drug use. Beverly Hills, CA; Sage.

[37] Gibbons F., Gerrard M., Vande Lune L., Wills T., Brody G., & Conger R. (2004). Context and cognitions: Environmental risk, social influence, and adolescent substance use. Personality and Social Psychology Bulletin, 30, 1048–1061. 10.1177/0146167204264788 15257788

[38] SAMHSA. (1997). National Household Survey on Drug Abuse.

[39] Ellis, L. K., & Rothbart, M. K. (2001, April). Revision of the Early Adolescent Temperament Questionnaire. Poster presented at the biennial meeting of the Society for Research in Child Development, Minneapolis, MN.

[40] Shaffer D. Fisher P., Lucas C. P., Dulcan M. K., & Schwab-Stone M. E. (2000). NIMH Diagnostic Interview Schedule for Children Version IV (NIMH DISC-IV): Description, differences from previous versions, and reliability of some common diagnoses. Journal of the American Academy of Child & Adolescent Psychiatry, 39 (1), 28–38. 10638065 10.1097/00004583-200001000-00014

[41] Thornberry, Terence P. The Use of Self-Reported Measures of Delinquency in Longitudinal Studies. NATO Advanced Workshop on Self-Reported Measures of Delinquency, Congress Centre, The Netherlands, 6 1988.

[42] Pillen M. B., & Hoewing-Roberson R. C. (1992). Determining Youth Gang Membership: Development of a Self-Report Instrument.

[43] Elliott D. S. (1990). National Youth Survey. Institute of Behavioral Science. University of Colorado

[44] Small S. A., & Kerns D. (1993). Unwanted sexual activity among peers during early and middle adolescence: Incidence and risk factors. Journal of Marriage & Family, 55, 941–952.

[45] Clark D. A., Donnellan M. B., Robins R. W., & Conger R, D. (2015). Early adolescent temperament, parental monitoring, and substance use in Mexican-origin adolescents. Journal of Adolescence, 41, 121–131. 10.1016/j.adolescence.2015.02.010 25841175 PMC4420705

[46] Curran P. J., Howard A. L., Bainter S. A., Lane S. T., & McGinley J. S. (2014). The separation of between-person and within-person components of individual change over time: A latent curve model with structured residuals. Journal of Consulting and Clinical Psychology, 82(5), 879 10.1037/a0035297 24364798 PMC4067471

[47] Berry D., & Willoughby M. T. (2017). On the practical interpretability of cross‐lagged panel models: Rethinking a developmental workhorse. Child Development, 88(4), 1186–1206. 10.1111/cdev.12660 27878996

[48] Wu W., Selig J. P., & Little T. D. (2013). Longitudinal data analysis. Oxford handbook of quantitative methods, 2, 387–410.

[49] Wu W., & Lang K. M. (2016). Proportionality assumption in latent basis curve models: A cautionary note. Structural Equation Modeling: A Multidisciplinary Journal, 23(1), 140–154.

[50] Curran P. J., Lee T., Howard A. L., Lane S., & MacCallum R. (2012). Disaggregating within-person and between-person effects in multilevel and structural equation growth models In Harring J. R. & Hancock G. R. (Eds.), CILVR series on latent variable methodology. Advances in longitudinal methods in the social and behavioral sciences (pp. 217–253). Charlotte, NC, US: IAP Information Age Publishing.

[51] Muthen L. K., & Muthen B. O. (1998–2018). Mplus user’s guide. Eighth Edition Los Angeles, CA: Muthen & Muthen.

[52] Allison P. D. (2009). Missing Data In Millsap R. E., & Maydeu-Olivares A. (Eds.), The SAGE Handbook of Quantitative Methods in Psychology (pp. 72–89). London: SAGE Publications Ltd.

[53] Falk C. F. (2018). Are Robust Standard Errors the Best Approach for Interval Estimation With Nonnormal Data in Structural Equation Modeling?. Structural Equation Modeling: A Multidisciplinary Journal, 25(2), 244–266.

[54] Hilbe J. M. (2011). Negative binomial regression. Cambridge University Press.

[55] Coxe S., West S. G., & Aiken L. S. (2009). The analysis of count data: A gentle introduction to poisson regression and its alternatives. Journal of Personality Assessment, 91(2), 121–136. 10.1080/00223890802634175 19205933

[56] Hancock G. R., & Liu M. (2012). Bootstrapping standard errors and data-model fit statistics in structural equation modeling.

